# *E. coli* gene regulatory networks are inconsistent with gene expression data

**DOI:** 10.1093/nar/gky1176

**Published:** 2018-11-20

**Authors:** Simon J Larsen, Richard Röttger, Harald H H W Schmidt, Jan Baumbach

**Affiliations:** 1 Department of Mathematics and Computer Science, University of Southern Denmark, Campusvej 55, 5230 Odense, Denmark; 2 Department of Pharmacology and Personalised Medicine, MaCSBio, Maastricht University, Universiteitssingel 60, 6229 ER, Maastricht, The Netherlands; 3 Chair of Experimental Bioinformatics, Wissenschaftszentrum Weihenstephan, Technical University of Munich, Maximus-von-Imhof-Forum 3, 85354 Freising-Weihenstephan, Germany

## Abstract

Gene regulatory networks (GRNs) and gene expression data form a core element of systems biology-based phenotyping. Changes in the expression of transcription factors are commonly believed to have a causal effect on the expression of their targets. Here we evaluated in the best researched model organism, *Escherichia coli*, the consistency between a GRN and a large gene expression compendium. Surprisingly, a modest correlation was observed between the expression of transcription factors and their targets and, most noteworthy, both activating and repressing interactions were associated with positive correlation. When evaluated using a sign consistency model we found the regulatory network was not more consistent with measured expression than random network models. We conclude that, at least in *E. coli*, one cannot expect a causal relationship between the expression of transcription and factors their targets, and that the current static GRN does not adequately explain transcriptional regulation. The implications of this are profound as they question what we consider established knowledge of the systemic biology of cells and point to methodological limitations with respect to single omics analysis, static networks and temporality.

## INTRODUCTION

The uncovering of genome-wide gene regulatory networks is a key tool in systems biology allowing researchers to understand the complex mechanisms of transcriptional gene regulation and to model the expressional behavior of genes in accordance to changing internal and environmental conditions. Collectively, these models form the basis of our current understanding of the complex systems biology of cells and multicellular organisms. The inference of regulatory interactions through computational methods has become a standard tool and with the rapidly growing body of available gene expression studies currently available, the reconstruction of transcriptional gene regulatory networks (GRNs) using gene expression data is becoming more and more promising.

The problem of inferring a gene regulatory network from gene expression data has received significant attention. It was the focus of four separate DREAM challenges, with DREAM5 in 2010 being the most recent one. A wide array of gene expression-based network inference methods have been developed (1,2). Despite these efforts, previous evaluations found that gene expression-based inference methods achieve very modest performance when applied to real data, despite performing well on *in silico* generated data (2,3). Additional methods incorporate multiple types of data such as network topology, sequence information and gene set enrichment (4–8) to improve predictive performance over a purely gene expression-based approach. Gene regulatory networks have commonly been modeled using Boolean networks (9,10), Bayesian networks (11,12) and ordinary differential equations (13–16). However, given that currently available regulatory information is provided as binary interaction networks, it is important to assess the validity of such networks regardless of the modeling approach taken.

The consistency between GRNs and gene expression studies has previously been studied in *E. coli* for a set of well-studied genes under four different conditions (17). The sign consistency model introduced by Siegel *et al.* (18) provided a mathematical framework for evaluating inconsistencies in signed interaction graphs. Several methods, often Cytoscape (19) plugins, have subsequently been developed for automating the detection of inconsistencies based on the sign consistency model. COMA (20) uses a Boolean network model to detect inconsistent interactions in the network with respect to a single expression study; BioQuali (21) detects inconsistent genes in the network and can suggest changes to the expression profile in order to better explain the experimental data; CytoASP (22) uses an answer set programming approach to identify inconsistent genes and suggest possible ways to repair them, either by changing expression profiles, changing the influence of an interaction, or by introducing new interactions. Finally, the SigNetTrainer tool (23) uses an integer linear programming approach to repair inconsistent signs in a single gene expression experiment to match a given interaction network, or identify insertions and deletions of interactions in the network to best match a given set of experiments.

Informed by our current understanding of transcriptional regulation it is generally assumed that an up- or downregulation of the genes coding for a transcriptional factor will result in a corresponding change in the expression of the genes regulated by that transcription factor. To investigate this assumption, we analyzed the consistency between a state of the art experimentally validated gene regulatory network of *E. coli* and a large compendium of gene expression profiles. We aimed to quantify how well the current gene regulatory network of *E. coli* corresponds to measured gene expression levels, and how well the transcriptional influence of TFs is reflected in the expression of their target genes. Our analysis revealed that both activating and repressing interactions were associated with positive correlation, which directly contradicted our preunderstanding. Furthermore, we show that, when evaluated using a sign consistency model, the regulatory network is not more consistent with measured gene expression than random network models. This implies that one cannot expect to see a causal relationship between the expression of transcription factors and their targets when evaluating the cell at a system-wide level.

## MATERIALS AND METHODS

### Data set preparation

We obtained a compendium of *E. coli* gene expression data from the DREAM5 challenge data set (3). The compendium contained expression for 4297 genes from 805 samples, all obtained with the Affymetrix *E. coli* Antisense Genome Array. The compendium contains expression from a wide range of experients including wild type, drug perturbations, environmental perturbations, gene knockout/knockdown and time series. The expression data was normalized using Robust Multi-array Averaging (RMA) and quantile normalization then log-scaled.

We obtained a set of regulatory interactions from the RegulonDB database (24), consisting of 4564 experimentally validated transcription factor (TF) to gene interactions and 2154 TF to transcription unit (TU) interactions. A transcriptional gene regulatory network was constructed as a directed bi-partite graph containing a vertex for each TF, and one for each gene and TU, respectively. An edge between pairs of vertices (TF to gene/TU) was added for each transcriptional interaction reported in RegulonDB. Each interaction in the regulatory network was labeled as either an activation (↑) or repression (↓). Interactions with dual or unknown regulatory effect were removed (0.4% and 2%, respectively). Annotation of 214 transcription factors and 1036 transcription units were also obtained from from RegulonDB. TFs and TUs where one or more of its constituent genes had no expression profile were removed from the network (12% and 24%, respectively). The resulting network contained 175 TFs, 1865 targets and 4503 interactions, where 54% of interactions were activating and the remaining 46% were repressing.

Genes in a TU are regulated together and thus we expect them to have similar expression. Because this is not always the case, we defined the expression of a TU as the mean expression of its constituent genes for each sample. For transcription factor complexes (i.e. several genes coding for proteins acting as TF complex together) we defined the expression level as the minimum level among the genes for each sample.

For comparison we also obtained an *in silico* generated data set from the DREAM5 challenge containing a simulated regulatory network and corresponding gene expression compendium. The *in silico* regulatory network was generated to have topology similar to known regulatory networks of model organisms using the method described in (25). The simulated gene expression data was generated based on the regulatory network using GeneNetWeaver (26). The generated data simulates wild types, as well as drug/environmental perturbations, knockout/knockdown experiments and time series. The *in silico* network contained 178 TFs, 1498 targets and 4012 interactions, where 56% of interactions were activating and the remaining 44% were repressing.

### Inconsistency detection

We evaluated the consistency between the regulatory networks and the gene expression compendia using a sign consistency model. We first computed contrasts from raw expression values in order to identify genes that were up- or downregulated in each experiment with respect to some reference. For each experiment in each compendium we identified an unperturbed sample to use as reference. Contrasts were then computed as the log-ratio between the reference and each other case in the experiment. In both the *E. coli* and *in silico* data set this resulted in 655 contrasts.

To assess the consistency between the regulatory network and the gene expression studies we identified a set of cases that we define to be *inconsistent*. We used an inconsistency model similar to the models used in BioQuali and COMA, but extended to include rules for genes that are unchanged. Each vertex (TF, gene or TU) was labeled as either upregulated (+), downregulated (−) or unchanged (0) for each contrast in the data set. Vertices were labeled according to the log-ratio between case and control and some threshold *t*, where vertices were considered downregulated when the log-ratio was less than −*t*, upregulated when greater than *t* and unchanged otherwise.

Intuitively, when a TF (or TF complex) is upregulated, we expect its target genes (or TUs) to be upregulated if it is an activator, and downregulated if it is a repressor. Similarly, if a TF is downregulated, we expect its target genes to not be upregulated if it is an activator, and not downregulated if it is a repressor. When the TF is unchanged the interaction is considered consistent regardless of the labeling of the target gene/TU and type of interaction. When the target gene/TU is unchanged, we consider an interaction inconsistent if the TF is upregulated and consistent otherwise. One could make this model more strict by defining all interactions where one but not both interactors are unchanged to be inconsistent, however, it is not well-defined which of these cases are actually inconsistent. For this reason we chose a more conservative definition in order to reduce the number of interactions falsely labeled as inconsistent. Table 1 gives a overview of which interactions were considered consistent and inconsistent.

**Table 1.** Overview of regulatory interactions considered inconsistent according to the expression of the transcription factor and target genes.

Regulation	TF exp.	Target exp.	Consistent
↑	+	+	yes
↑	+	−	no
↑	+	0	no
↑	−	+	no
↑	−	−	yes
↑	−	0	yes
↓	+	+	no
↓	+	−	yes
↓	+	0	no
↓	−	+	yes
↓	−	−	no
↓	−	0	yes
↑, ↓	0	+, −, 0	yes

The symbols in column 1 describe whether the interaction is an activation (↑) or repression (↓).

The symbols in columns 2 and 3 signify whether the vertex is labeled upregulated (+), downregulated (−) or unchanged (0).

Let the *inconsistency vector* of an edge *e* be a vector *I*(*e*) ∈ {0, 1}^*n*^, where *n* is the number of contrasts and *I*_*i*_(*e*) = 1 if *e* is inconsistent with respect to contrast *i* and *I*_*i*_(*e*) = 0 otherwise. Let the inconsistency vector of a gene or TU $v$ be a vector *J*(*v*) ∈ {0, 1}^*n*^, where

**Figure 1.**
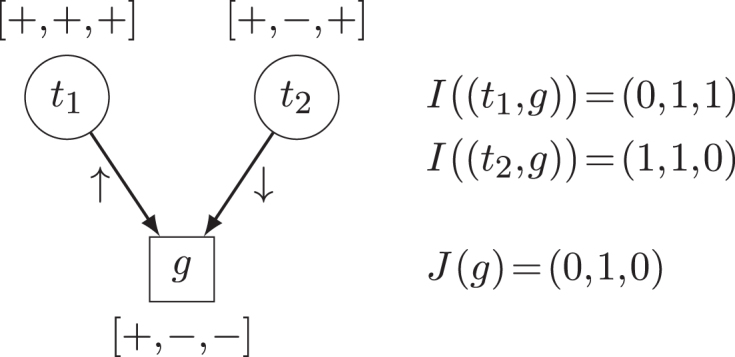
Example of consistency vectors for a gene *g* regulated by two transcription factors *t*_1_ and *t*_2_. Labels for three different contrasts are shown in brackets next to the vertices.

(1) \begin{equation*} J_i(v) = \prod _{e \in N^-(v)} I_i(e), \end{equation*}

and *N*^−^(*v*) is the set of incoming edges of *v*. When a target gene/TU is subject to multiple regulators, it is considered consistent with respect to a specific contrast if there exists at least one incoming regulation that is consistent in that contrast. If several TFs of different types (i.e. activators and repressors) regulate the same target gene/TU, it is generally unclear which regulation is the dominant one. In our case we are looking for a conservative lower bound (i.e. the minimum inconsistency load) and resolve such cases by marking a gene/TU as ‘explained’ if any regulator explains its over-/underexpression. We define the number of inconsistencies for an interaction *e* as |*I*(*e*)|_1_. Similarly, we define the number of inconsistencies for a vertex *v* as |*J*(*v*)|_1_ (Figure 1). Furthermore, we define the *global inconsistency load* for a network as the total number of inconsistencies among all genes and TUs in the network.

### Network perturbation methods

In order to assess the consistency of our regulatory network compared to random data (the null model), two methods of perturbation were implemented. The broad distribution of node degrees suggests that the degree is an important characteristic for nodes in biological networks (27). For this reason, both perturbation methods preserve the degree of all nodes in the network to rule out the impact of node degree distribution on consistency.

The first method perturbs the expression data by uniformly redistributing the entire expression profiles of genes using a Fisher-Yates shuffle. This method produces a regulatory network with identical topology to that of the original network, but with randomly assigned expression profiles. Entire profiles are exchanged in order to keep each profile internally consistent. If individual expression values of different genes were to be exchanged instead, we would no longer quantify how consistent a random set of interactions is wrt. the gene expression compendium, but instead how consistent random gene expression is wrt. the regulatory network.

The second method perturbs the topology of the network while preserving both the in-degree and out-degree all nodes. This is achieved using a simple numerical algorithm proposed in (28). The algorithm selects two existing edges (*p, q*) and (*r, s*), and rewires their endpoints such that they become two new edges, (*p, s*) and (*r, q*). If one or both of these new edges already exist, the procedure is aborted and another pair is selected instead. This procedure is repeated 10 · |*E*| times, where |*E*| is the number of edges in the network. This method produces a network with a randomly perturbed topology but identical node degree distribution to that of the original network, while preserving the expression profiles of individual genes.

## RESULTS

### Correlation of expression profiles

**Figure 2.**
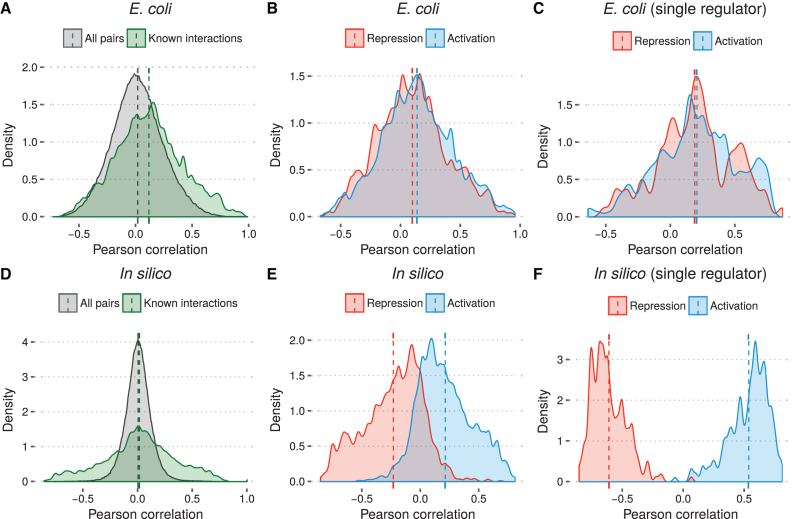
Distribution of Pearson correlation coefficients for TF and target gene/unit pairs. (**A**, **D**) Comparison between correlation of all possible TF-target pairs and all known interactions. (**B**, **E**) Comparison between correlation of known activations and repressions. (**C, F**) Comparison between correlation of known activations and repressions where the TF is the only regulator of the target. Dashed vertical lines indicate mean correlation for each set of interactions.

First we assessed the correlation between gene expression profiles of known TF and target gene (or TU) interactions in the regulatory network of *E. coli*. The correlation was determined using Pearson’s correlation coefficient. We first examined the distribution of correlations for known TF-target pairs and observed a mean correlation of 0.12 (Figure 2A). When evaluating all possible TF-target pairs in the network we observed a mean correlation of 0.02. Based on the assumption that greater expression of a repressor will lead to a lower expression of its targets, one would expect the the repressors to be associated with anti-correlation. However, when separating the interactions by regulatory influence, we observe a modest positive correlation in both cases (0.10 and 0.14 for repression and activation, respectively) (Figure 2B).

When looking at the correlation of interactions in isolation, we do not account for more complex relationships such as multiple TFs regulating the same gene. To investigate whether this has an impact, we examined the distribution of correlations for only those interactions where the TF is the sole regulator of the target gene or TU (17% of interactions, 40% of targets). For this reduced set of interactions, we observe a similar distribution, but with a greater mean correlation for both types of interactions (0.19 and 0.20 for repressors and activators, respectively) (Figure 2C). Surprisingly, for single-regulator interactions the difference in mean correlation between activations and repressions was in fact smaller than for the complete regulatory network.

We repeated this evaluation for the *in silico* data set and found highly dissimilar results. The mean correlation of known interactions was close to zero (0.02), but with high variance compared to the background distribution (Figure 2D). When separating interactions by type we observed a clear separation between the two distributions, with repressors overall associated with negative correlation and activators associated with positive correlation (mean –0.23 and 0.21, respectively) (Figure 2E). When considering only single-regulator interactions we observed an even stronger separation between the distributions with almost no overlap (Figure 2F).

### Inconsistency between regulatory network in expression data

We used an inconsistency model to evaluate how consistent the regulatory network was with the gene expression data. We use a threshold of ±0.043 for the *E. coli* data and ±0.26 for the *in silico* data. These threshold were chosen such that ≈50% of contrast values were considered either up- or downregulated in the model. We first compared the global inconsistency load (total number of inconsistent cases among all targets) of the *E. coli* regulatory network to two random network models. The first method perturbs the network by random edge rewiring, while the second method redistributes gene expression profiles among all genes (see Methods). For each perturbation method the experiment was repeated 200 times.

**Figure 3.**
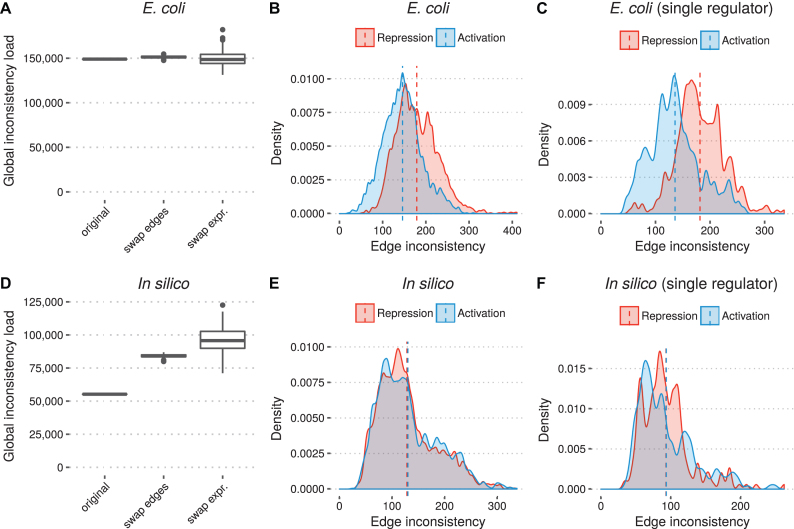
Evaluation of inconsistency load in regulatory network and perturbed network models. (**A**, **D**) Global inconsistency load in regulatory networks compared to two random networks models. For the random models, each experiment was repeated 200 times. (**B**, **E**) Distribution of edge inconsistency for repressing and activating interactions. (**C**, **F**) Distribution of edge inconsistency for repressing and activating interactions targeting genes/TUs with only one regulator. Dashed vertical lines in (B, C, E, F) indicate mean inconsistency for each set of interactions.

The global inconsistency load for the unperturbed network was 148 959. When perturbing the edges of the network the median global inconsistency load increased to 151 293 (1.6% increase) and when perturbing the expression profiles instead, the median inconsistency load decreased to 148 584 (0.3% decrease) (Figure 3A). The high similarity between the consistency load of the regulatory network and the null models suggests that the regulatory network is not significantly more consistent with the expression data than a random network. In fact, and surprisingly, when redistributing the expression profiles a majority of cases were more consistent than the unperturbed network. When separating the interactions by type we observed that repressive interactions were on average more inconsistent (mean 178.7) than activating interactions (mean 145.9) (Figure 3B). For single-regulator targets the mean inconsistency was slightly higher for repression and slightly lower for activation (181.1 and 135.5, respectively) (Figure 3C).

In the *in silico* data, we observed that perturbing the network greatly increased the overall inconsistency. The global inconsistency load for the unperturbed network was 55 247, increasing to median 84041.5 (52% increase) when rewiring interactions and median 95 710 (73% increase) (Figure 3D). When separating interactions by type, the mean number of interactions is nearly identical (128.2 and 130 for repression and activation, respectively) (Figure 3E). This was also the case when considering only single-regulator targets (Figure 3F).

### Association between inconsistency and experimental evidence

**Figure 4.**
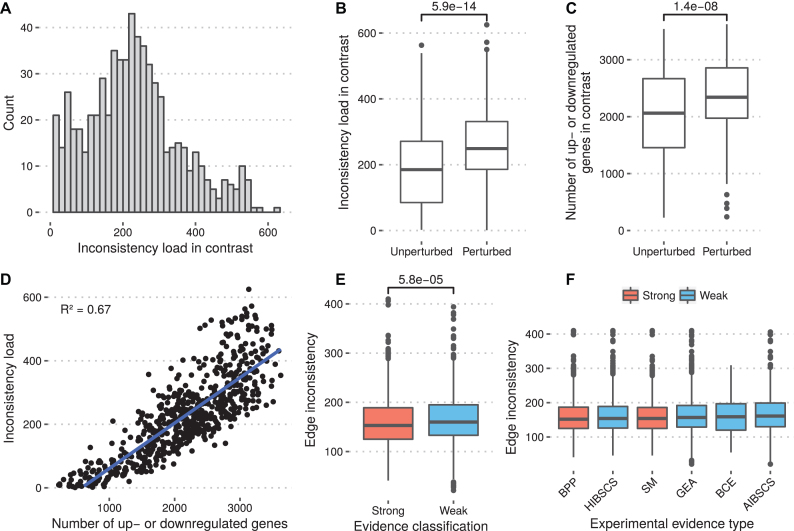
Evaluation of inconsistency load of *E. coli* across contrasts and experimental evidence types. (**A**) Distribution of inconsistency load across the 655 contrasts. (**B**) Comparison between inconsistency load in contrasts with and without perturbation (e.g. drugs and experimental contitions). (**C**) Comparison between number of up- or downregulated genes in sign consistency model for in contrasts with and without perturbation. (**D**) Relationship between number of up- or downregulated genes and inconsistency load in contrasts. (**E**) Comparison between inconsistency of interactions with strong and weak experimental evidence. (**F**) Comparison between different common experimental evidence types for regulatory interactions. Evidence types: binding of cellular extracts (BCE), site mutation (SM), binding of purified proteins (BPP), gene expression analysis (GEA), human inference based on similarity to consensus sequences (HIBSCS), automated inference based on similarity to consensus sequences (AIBSCS). Significance in (B, C, E) was computed using a Mann–Whitney *U*-test.

We examined the inconsistency load for each of the 655 contrasts to determine if some experimental conditions were associated with higher inconsistency. The number of inconsistencies ranged from 2 to 625, with mean 227.4 (Figure 4A). We observed that contrasts where the case condition was subjected to a perturbation (e.g. drugs or environmental perturbations) were associated with a greater inconsistency load (mean 267.9 versus 191.2, Mann–Whitney *U* *p* = 5.9e−14) (Figure 4B). However, we further observed that the perturbed experiments were associated with a higher variance in fold change and consequently a greater number of genes marked up- or downregulated in the sign consistency model (Figure 4C) and that the number of up- or downregulated genes in a contrast was highly correlated with number of inconsistencies which may explain this discrepancy (Figure 4D).

We also examined whether some interactions reported in RegulonDB were more inconsistent with the expression data than others. All interactions in RegulonDB are classified based on the type and amount of experimental evidence reported in the database. We observed that the mean inconsistency for interactions with strong experimental evidence was marginally lower than interactions with only weak evidence (mean 159.0 versus 163.7, Mann–Whitney *U* *p* = 5.8e−5) (Figure 4E). We further evaluated the overall inconsistency based on the type of experimental evidence (excluding uncommon methods, occurring <100 times). Out of the six evidence types considered, the two methods considered strong evidence by RegulonDB, namely *binding of purified proteins* and *site mutation*, ranked first and third in consistency. However, mean inconsistency was very similar across methods, ranging from 158.5 to 164.0 (Figure 4F).

## DISCUSSION

We investigated, in *E. coli*, the commonly held assumption, that a change in the expression of a transcription factor should have a positive or negative causal effect on the expression of its targets, depending on the type of regulation. This behavior was, however, not observed. Instead we on average observed a positive correlation between transcription factors and their targets, regardless of their regulatory influence, with similar distributions for both interaction types. In many cases a gene or TU is regulated by several transcription factors. For such targets, it may be too simplistic to expect each interaction to be reflected in the mRNA levels. However, when looking at only regulations of genes/TUs with only one regulator, we observed a similar pattern, again with repressive interactions being positively correlated. The set of regulatory interactions reported in RegulonDB is most likely far from complete, and thus some of the targets considered as having a single regulator may in fact be regulated through other undiscovered interactions. However, this does not explain why the mean correlation coefficient for repression was both greater and more similar to activation for single-regulator targets.

As expected, no correlation between random pairs of transcription factors and genes/TUs was observed. This suggests that the correlation we observe in the unperturbed data is a property of the network and not simply because the gene expression experiments are correlated (e.g. genes having a general, experiment-specific chance of being more upregulated in one experiment than another one). In this study we measured correlation using the Pearson correlation coefficient. It could be the case that there is a causal but non-linear relationship between the expression of TFs and their targets, which would not be captured with Pearson correlation. To confirm this was not the case, we repeated the correlation analysis using Spearman’s rank correlation coefficient which produced analogous results (Supplementary Figure S1).

When comparing the total inconsistency load of the GRN to random network models, we observed a remarkably similar degree of consistency. This suggests that the experimentally validated network does not explain the transcriptome data better than a random network, at least with respect to our sign consistency model. Repressive interactions were on average associated with a higher degree of inconsistency than activating interactions. This is not surprising, given that the underlying assumption that repressors and their targets should be anti-correlated is contrary to what was observed in real gene expression.

The choice of fold change threshold for when genes are considered up- or downregulated may have a significant impact on the results. To investigate this we repeated the inconsistency analysis with a lower and higher threshold (Supplementary Figure S2–S5). We observed that a lower threshold resulted in a higher consistency load, but the overall patterns were the same: the global inconsistency load in *E. coli* was mostly unchanged under random perturbation, but increased significantly in the *in silico* data, and repressive interactions were on average more inconsistent in *E. coli* but almost identical to activating interactions in *in silico*.

In our signed model TFs, genes and TUs were classified as either upregulated, downregulated or unchanged. Previous methods have generally used a binary classification where genes were simply labeled ‘up’ and ‘down’ (or ‘present’ and ‘not present’), but we included the unchanged state to avoid small changes in expression being considered a change in regulation. We implemented a binary consistency model as well and repeated the inconsistency evaluation for different thresholds (Supplementary Text S1.1). We observed that perturbing the network has little effect on the overall inconsistency in *E. coli* but greatly increases inconsistency in the *in silico* data (Supplementary Figure S6–S8). These results confirm that the poor consistency we observed in this study is not a result of our model being too conservative.

We acknowledge that our model for evaluating the inconsistency is both simplistic and conservative. However, the stark contrast between the *E. coli* and *in silico* data and the fact that perturbing the *in silico* data greatly increased the inconsistency load demonstrates, that our model should be able to recognize it if the expression data more closely resembled the idealized model emulated in the *in silico* data.

Several possible explanations exist for the observed lack of a causal relationship between TFs and targets. In previous studies on *E. coli* and *S. cerevisiae*, the regulatory response of target genes has, in some cases, been observed to be time delayed (29). When this is the case, a regulatory response cannot be detected by correlating expression of genes at the same time points. To determine such relationships, a time series analysis is necessary. The lack of correlation could also suggest that many TFs are not significantly transcriptionally regulated (30). If a TF is primarily regulated by post-translational modifications such as phosphorylation by two-component signaling (31,32), its activity not will be reflected significantly in the expression level of its coding gene. Another reason for the poor correlation of regulator/target could be a generally modest correlation of transcriptome and proteome data (33,34). In addition, even if a TF is transcribed and translated, it may be in an inactive conformation. The lack of correlation may thus also be due to TFs not bound by their activator metabolites, or bound by inactivators, both of which cannot be observed from transcriptome data.

While these results may be surprising, they are well in line previous performance evaluations. In the DREAM5 challenge, even the best performing methods did not achieve high precision beyond very low recall values despite performing well on the *in silico* data (3). A similar result was reported by Madhamshettiwar *et al.* (2) who compared several methods on *in silico* data and microarray gene expression from ovarian cancer patients. We argue that our results bring into question the applicability of previously published methods based on the detection of inconsistencies between known regulations and gene expression, and possibly even methods based on correlation or mutual information as well. This does not imply that inconsistency detection cannot be utilized to produce meaningful results, but it does suggest that more sophisticated models are needed, for instance, involving mult-omics, more diverse network types, or temporality. Finally, the clear difference between the real and synthetic expression data brings into question the validity of using simulated data for evaluating methods. If simulated data is to be used for evaluation going forward, one must ensure that the assumptions underlying the simulated model accurately reflect gene expression patterns *in vivo*.

## CONCLUSION

We evaluated the overall consistency between the experimentally validated regulatory network of *E. coli* found in RegulonDB and a large compendium of microarray gene expression data. We observed that both activating and repressing interactions were associated with a positive correlation between the expression of transcription factors and their targets, and that the distributions of correlation for activating and repressing interactions were remarkably similar. When evaluated using a sign consistency model the regulatory network was not significantly more consistent with measured gene expression than two random network models. Our results suggest that one cannot expect a causal relationship between the expression of transcription factors and their targets and, as such, currently available static gene regulatory networks do not adequately explain transcriptional gene expression – at least not on a systems-wide level. As a way forward, we urge to researchers to reconsider this flawed view on the relationship between transcriptional regulation and gene expression, and we suggest to base future methods on more complex models, multi-omics data, using several network types and/or including temporality.

## Supplementary Material

Supplementary Data
