# Supplementary material for: E. coli gene regulatory networks are inconsistent with gene expression data
Source: Nucleic Acids Res. 2018 Nov 20;47(1):85–92. doi: 10.1093/nar/gky1176 (PMC6326786; doi:10.1093/nar/gky1176)
Supplement: Supplementary Data [file gky1176_supplemental_files.pdf]

Supplementary materials for: *E. coli* gene regulatory networks are inconsistent with gene expression data

Simon J. Larsen [1,*], Richard Röttger [1], Julio Collado-Vides [2], Harald H.H.W. Schmidt [3] and Jan Baumbach [1,4]

[1]Department of Mathematics and Computer Science, University of Southern Denmark, Campusvej 55, 5230 Odense, Denmark [2]Centro de Ciencias Genmicas, UNAM Campus Morelos, Av. Universidad s/n, Chamilpa, 62210 Cuernavaca, Mor., Mexico [3]Department of Pharmacology and Personalised Medicine, CARIM, Faculty of Health, Medicine & Life Science, Maastricht University, Maastricht, The Netherlands [4]Chair of Experimental Bioinformatics, Wissenschaftszentrum Weihenstephan, Technical University of Munich, Maximus-von-Imhof-Forum 3, 85354 Freising-Weihenstephan, Germany

S1 Supplementary text

S1.1 Binary consistency model

We implemented an alternative consistency model similar to the on implemented in COMA [1]. Each vertex in the network (TF, gene and TU) was labeled as either "present" (+) or "not present" (−) for gene expression experiment. A vertex was considered present if its absolute expression was above some threshold t and not present otherwise. Interactions are labeled as either activation (+) or repression (−) similarly to in the model described in the main manuscript. The consistency rules are outlined in Table S1. Let the *inconsistency vector* of an edge e be a vector $I(e) \in \{0,1\}^n$, where n is the number of contrasts and $I_i(e) = 1$ if e is inconsistent with respect to contrast i and $I_i(e) = 0$ otherwise. Let the inconsistency vector of a gene or TU v be a vector $J(v) \in \{0,1\}^n$, where

$$J_i(v) = \prod_{e \in N^-(v)} I_i(e), \tag{1}$$

and $N^-(v)$ is the set of incoming edges of v. When a target gene/TU is subject to multiple regulators, it is considered consistent with respect to a specific contrast if there exists at least one incoming regulation that is consistent with that contrast. Finally, as before, we define the *global inconsistency load* for a network as the total number of inconsistencies among all genes and TUs in the network.

*To whom correspondence should be addressed. Email: sjlarsen@imada.sdu.dk

Regulation	TF exp.	Target exp.	Consistent
$\uparrow$	$+$	$+$	yes
$\uparrow$	$+$	$-$	no
$\uparrow$	$-$	$+$	no
$\uparrow$	$-$	$-$	yes
$\downarrow$	$+$	$+$	no
$\downarrow$	$+$	$-$	yes
$\downarrow$	$-$	$+$	yes
$\downarrow$	$-$	$-$	no

Table S1: Overview of regulatory interactions considered inconsistent according to the expression of the transcription factor and target genes. The symbols in column 1 describes whether the interaction is an activation ($\uparrow$) or repression ($\downarrow$). The symbols in column 2 and 3 signify whether the vertex is labeled present ($+$) or not present ($-$).

S2 Supplementary figures

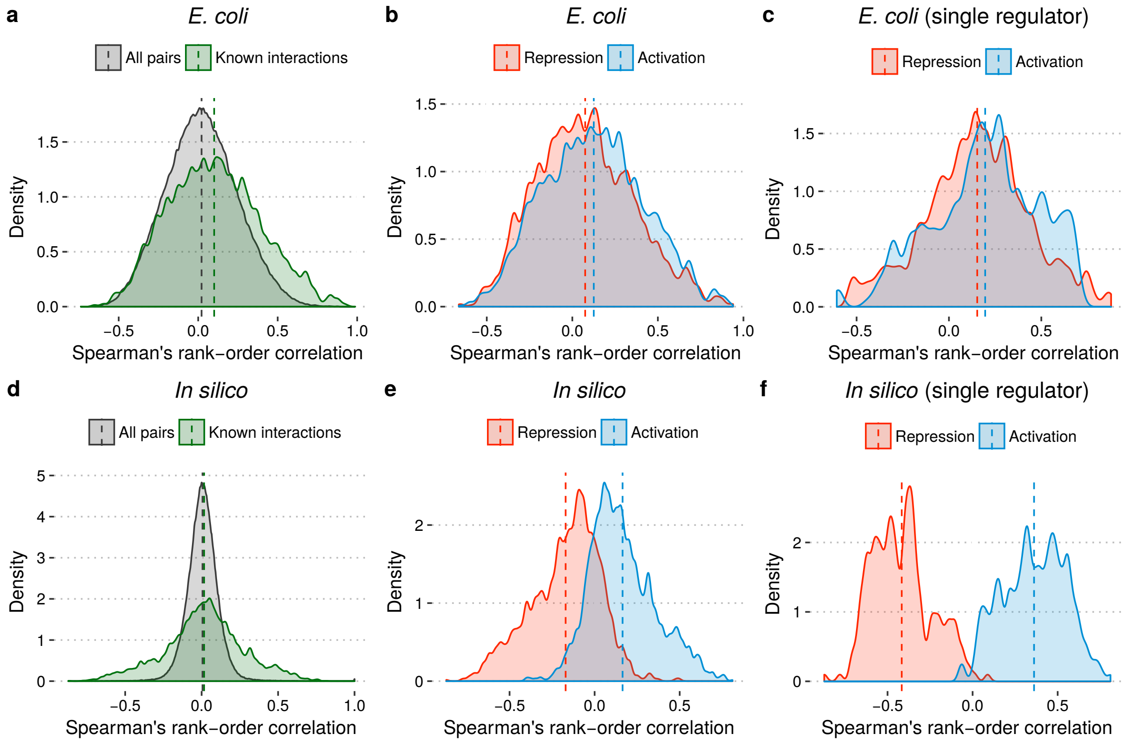

Figure S1: Distribution of Spearman's rank-correlation coefficients for TF and target gene/unit pairs. (a,d) Comparison between correlation of all possible TF-target pairs and all known interactions. (b,e) Comparison between correlation of known activations and repressions. (c,f) Comparison between correlation of known activations and repressions where the TF is the only regulator of the target. Dashed vertical line indicates mean correlation for each set of interactions.

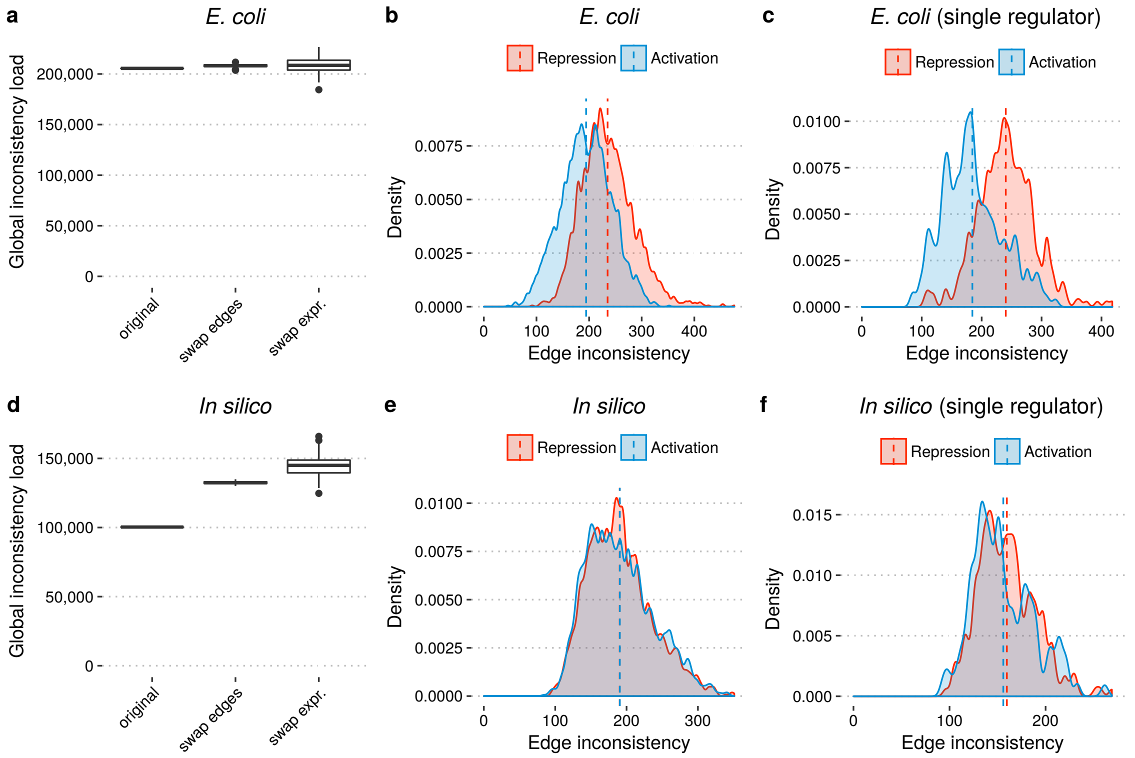

Figure S2: Evaluation of inconsistency load in regulatory network and perturbed network models. Log ratio cutoff for both data sets were selected such that 66% of contrast values were considered up- or downregulated. (a,d) Global inconsistency load in regulatory networks compared to two random networks models. For the random models, each experiment was repeated 200 times. (b,e) Distribution of edge inconsistency for repressing and activating interactions. (c,f) Distribution of edge inconsistency for repressing and activating interactions targeting genes/TUs with only one regulator. Dashed vertical line in (b,c,e,f) indicates mean for each set of interactions.

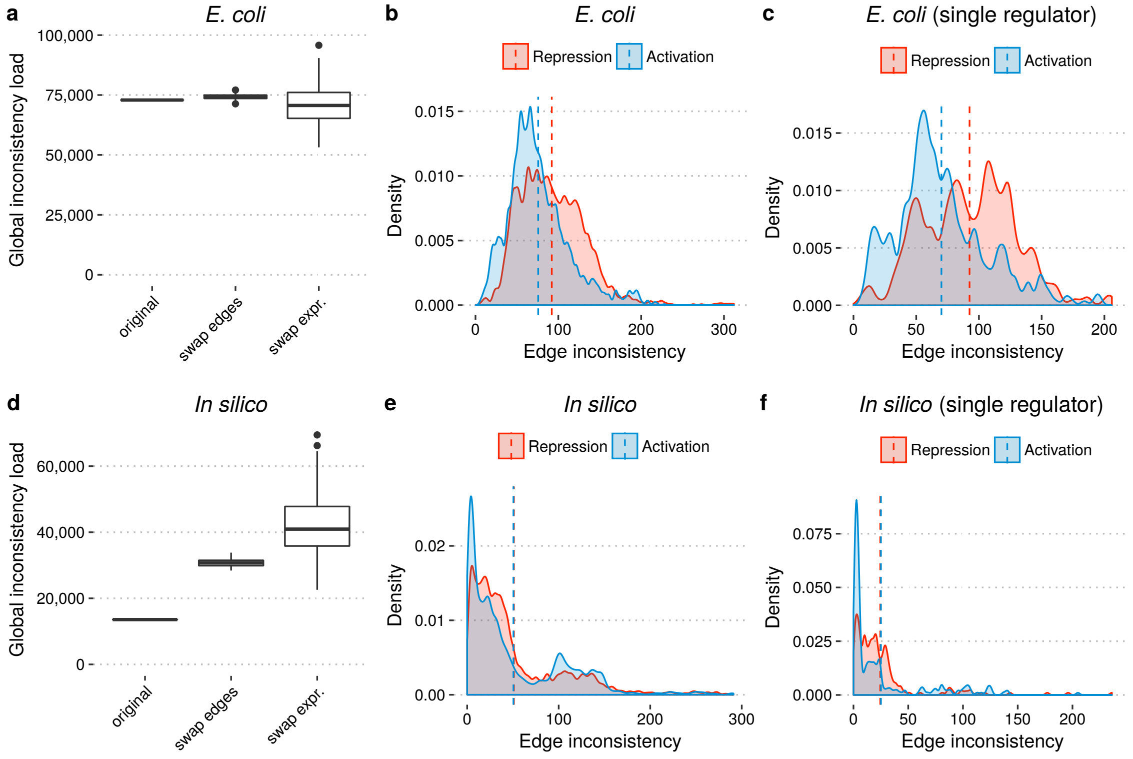

Figure S3: Evaluation of inconsistency load in regulatory network and perturbed network models. Log ratio cutoff for both data sets were selected such that 25% of contrast values were considered up- or downregulated. (a,d) Global inconsistency load in regulatory networks compared to two random networks models. For the random models, each experiment was repeated 200 times. (b,e) Distribution of edge inconsistency for repressing and activating interactions. (c,f) Distribution of edge inconsistency for repressing and activating interactions targeting genes/TUs with only one regulator. Dashed vertical line in (b,c,e,f) indicates mean for each set of interactions.

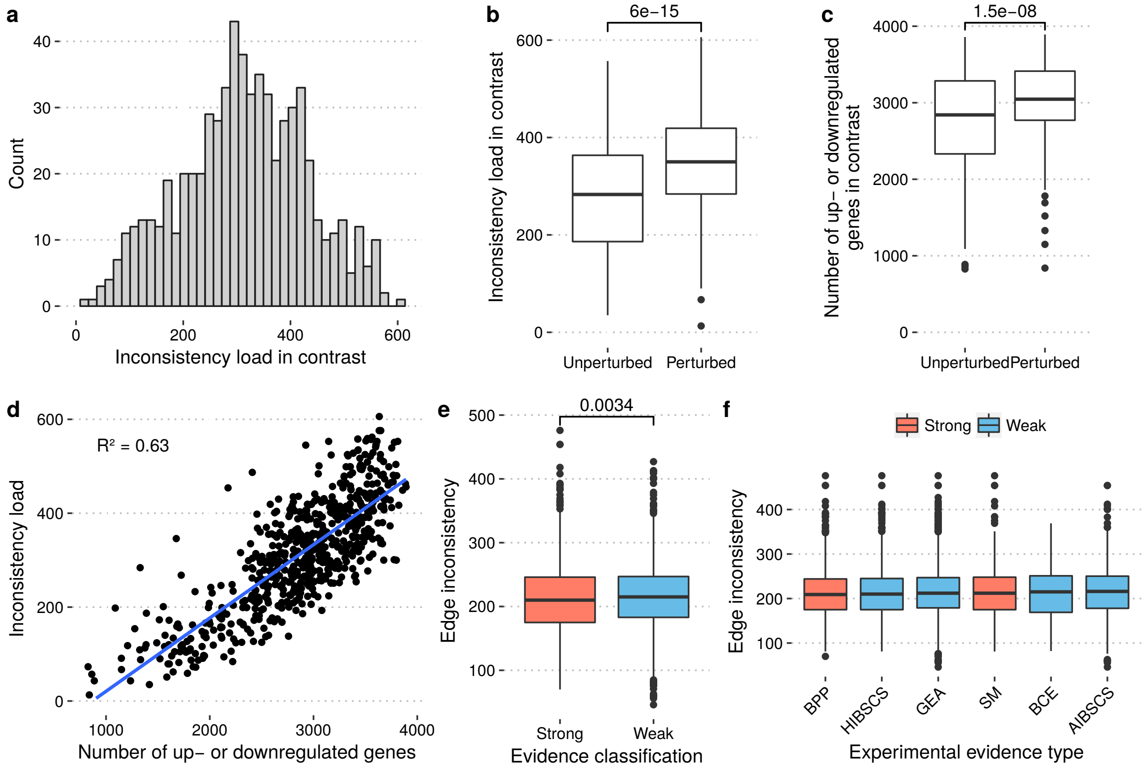

Figure S4: Evaluation of inconsistency load of *E. coli* across contrasts and experimental evidence types. Log ratio cutoff for both data sets was selected such that 66% of contrasts values were considered up- or downregulated. (a) Distribution of inconsistency load across the 655 contrasts. (b) Comparison between inconsistency load in contrasts with and without perturbation (e.g. drugs and experimental contitions). (c) Comparison between number of up- or downregulated genes in sign consistency model for in contrasts with and without perturbation. (d) Relationship between number of up- or downregulated genes and inconsistency load in contrasts. (e) Comparison between inconsistency of interactions with strong and weak experimental evidence. (f) Comparison between different common experimental evidence types for regulatory interactions. Evidence types: binding of purified proteins (BPP), human inference based on similarity to consensus sequences (HIBSCS), gene expression analysis (GEA), site mutation (SM), binding of cellular extracts (BCE), automated inference based on similarity to consensus sequences (AIBSCS). Significance in (b,c,e) computed using Mann-Whitney U-test.

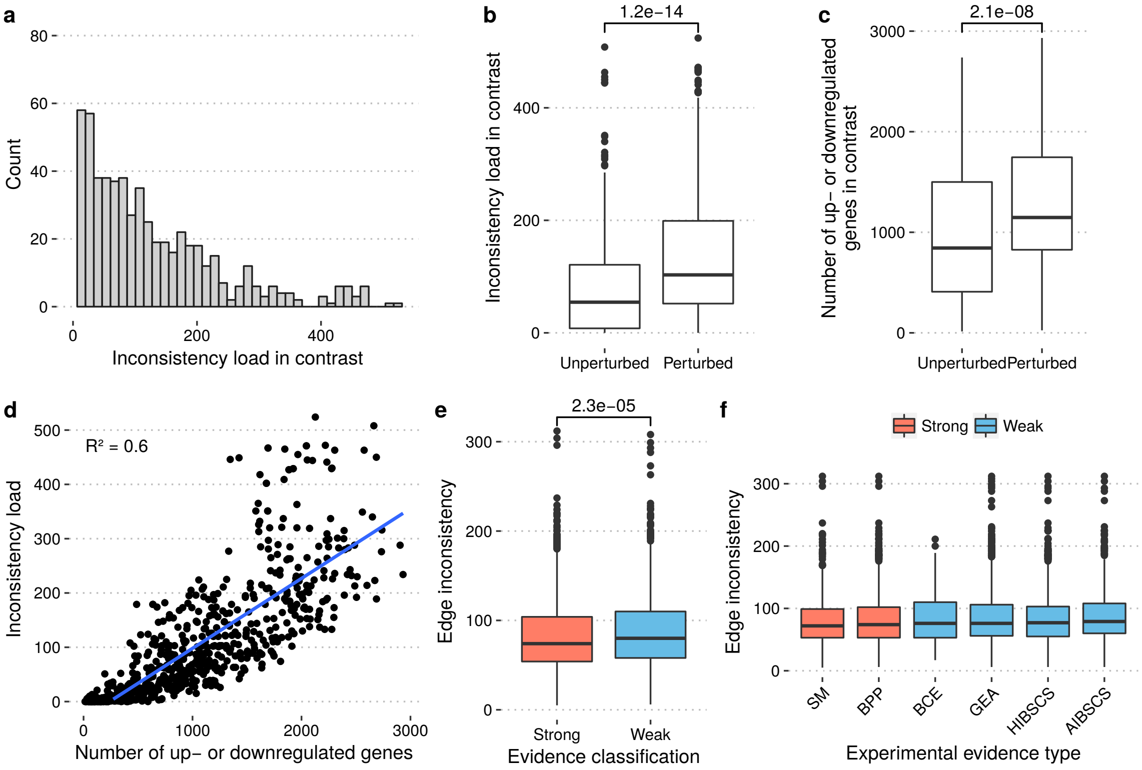

Figure S5: Evaluation of inconsistency load of *E. coli* across contrasts and experimental evidence types. Log ratio cutoff for both data sets was selected such that 25% of contrasts values were considered up- or downregulated. (a) Distribution of inconsistency load across the 655 contrasts. (b) Comparison between inconsistency load in contrasts with and without perturbation (e.g. drugs and experimental contitions). (c) Comparison between number of up- or downregulated genes in sign consistency model for in contrasts with and without perturbation. (d) Relationship between number of up- or downregulated genes and inconsistency load in contrasts. (e) Comparison between inconsistency of interactions with strong and weak experimental evidence. (f) Comparison between different common experimental evidence types for regulatory interactions. Evidence types: site mutation (SM), binding of purified proteins (BPP), binding of cellular extracts (BCE), gene expression analysis (GEA), human inference based on similarity to consensus sequences (HIBSCS), automated inference based on similarity to consensus sequences (AIBSCS). Significance in (b,c,e) computed using Mann-Whitney U-test.

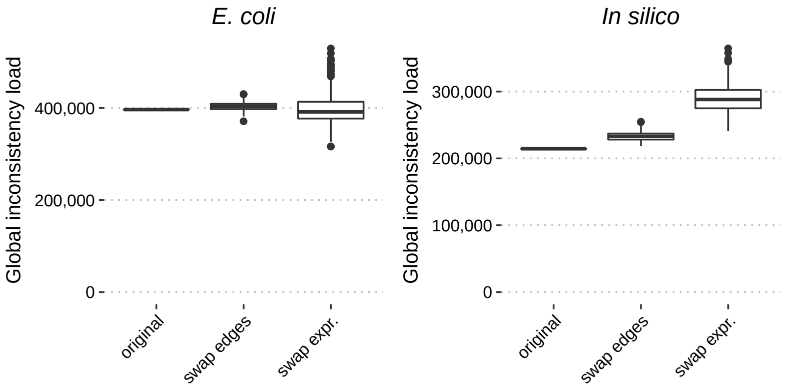

Figure S6: Global inconsistency load in regulatory networks compared to two random networks models using the binary consistency model. Genes were considered "present" if their expression was over the 13th percentile. For the random models, each experiment was repeated 200 times.

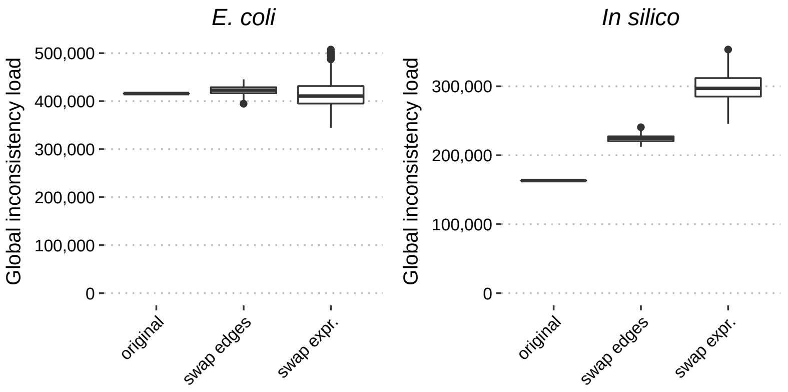

Figure S7: Global inconsistency load in regulatory networks compared to two random networks models using the binary consistency model. Genes were considered ”present” if their expression was above the 25th percentile. For the random models, each experiment was repeated 200 times.

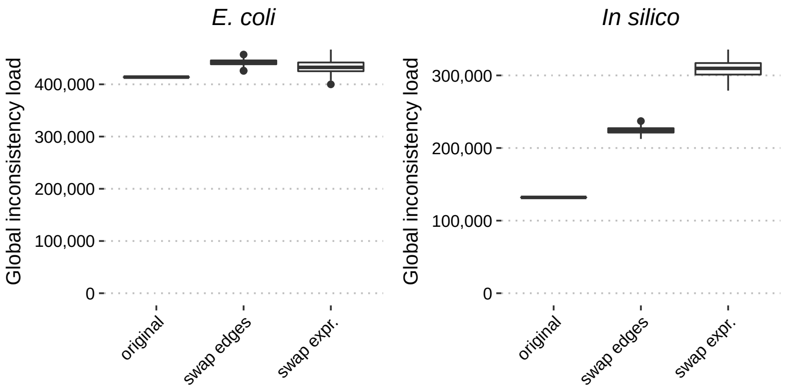

Figure S8: Global inconsistency load in regulatory networks compared to two random networks models using the binary consistency model. Genes were considered ”present” if their expression was above the 50th percentile. For the random models, each experiment was repeated 200 times.

References

[1] Jan Baumbach and Leonard Apeltsin. "Linking Cytoscape and the corynebacterial reference database CoryneRegNet". In: *BMC Genomics* 9.1 (2008), p. 184. DOI: 10.1186/1471-2164-9-184.
